# Gated Nanosensor for Sulphate-Reducing Bacteria Detection

**DOI:** 10.3390/nano15100774

**Published:** 2025-05-21

**Authors:** Alba López-Palacios, Ángela Morellá-Aucejo, Yolanda Moreno, Román Ponz-Carcelén, María Pedro-Monzonís, M. Dolores Marcos, Andrea Bernardos, Félix Sancenón, Elena Aznar, Ramón Martínez-Máñez, Andy Hernández-Montoto

**Affiliations:** 1 Instituto Interuniversitario de Investigación de Reconocimiento Molecular y Desarrollo Tecnológico (IDM), Universitat Politècnica de València, Universitat de València, Camino de Vera s/n, 46022 Valencia, Spain; aloppal1@upvnet.upv.es (A.L.-P.); ngemoau@upvnet.upv.es (Á.M.-A.); mmarcos@qim.upv.es (M.D.M.); anberba@upvnet.upv.es (A.B.); fsanceno@upvnet.upv.es (F.S.); anhermo4@doctor.upv.es (A.H.-M.); 2 CIBER de Bioingeniería Biomateriales y Nanomedicina, Instituto de Salud Carlos III, 46022 Valencia, Spain; 3 Unidad Mixta de Investigación en Nanomedicina y Sensores, Universitat Politècnica de València, Instituto de Investigación Sanitaria La Fe (IISLAFE), Av. Fernando Abril Martorell, 106, 46026 Valencia, Spain; 4 Instituto Universitario del Agua y Medio Ambiente (IIAMA), Universitat Politècnica de València, Camí de Vera s/n, 46022 Valencia, Spain; ymoren@upvnet.upv.es; 5 Global Omnium Group, Gran Vía Marqués del Turia, 19, 46005 Valencia, Spain; rponz@emimet.es (R.P.-C.); mapemo@globalomnium.com (M.P.-M.); 6 Unidad Mixta UPV-CIPF de Investigación en Mecanismos de Enfermedades y Nanomedicina, Universitat Politècnica de València, Centro de Investigación Príncipe Felipe, Avenida Eduardo Primo Yúfera, 3, 46012 Valencia, Spain; 7 Departamento de Química, Universitat Politècnica de València, Camí de Vera s/n, 46022 Valencia, Spain

**Keywords:** nanomaterials, molecular gates, sulphate-reducing bacteria, microbiologically influenced corrosion, oligonucleotide probe

## Abstract

*Desulfovibrio vulgaris* is an anaerobic microorganism belonging to the group of sulphate-reducing bacteria (SRB). SRB form biofilms on metal surfaces in water supply networks, producing a microbiologically influenced corrosion (MIC). This process produces the deterioration of metal surfaces, leading to high economic costs and different environmental safety and health problems related to its chemical treatment. For that reason, rapid and accurate detection methods of SRB are needed. In this work, a new detection system for *Desulfovibrio* has been developed using gated nanoporous materials. The probe is based on hybrid nanoporous alumina films encapsulating a fluorescent molecule (rhodamine B), whose release is controlled by an oligonucleotide gate. Upon exposure to *Desulfovibrio*’s genomic material, a movement of the oligonucleotide gatekeeper happens, resulting in the selective delivery of the entrapped rhodamine B. The developed material shows high selectivity and sensitivity for detecting *Desulfovibrio* DNA in aqueous buffer and biological media. The implementation of this technology for the detection of *Desulfovibrio* as a tool for monitoring water supply networks is innovative and allows real-time in situ monitoring, making it possible to detect the growth of *Desulfovibrio* inside of pipes at an early stage and perform timely interventions to reverse it.

## 1. Introduction

Microbiologically influenced corrosion (MIC) represents a significant deterioration process driven by arranged microorganisms forming a biofilm [1]. These microorganisms cause organic matter deposits on submerged metal surfaces, producing different corrosive gases that induce biocorrosion and several changes in the surface behaviour. As a result, metal surfaces degrade, leading to a marked reduction in their effectiveness and usable duration [2]. Furthermore, this process produces a new interface between the solution and the metal surface, potentially accelerating MIC. Thus, the economic costs and limited understanding of MIC make it an ongoing challenge. Moreover, the usual solutions to this problem involve the use of toxic and hazardous chemicals, which raise health and environmental safety concerns. MIC monitoring is challenging due to the diversity in biofilm composition and spatial variability. Hence, understanding the microorganisms involved in MIC processes and developing detection and treatment strategies are crucial [3].

Sulphate-reducing bacteria (SRB) are obligate anaerobes that stand out as the primary culprits in MIC. SRB derive electrons from hydrogen and various organic compounds (lactate, acetate, pyruvate, and malate), while employing sulphate as the terminal electron acceptor to produce hydrogen sulphide. This sulphate reduction makes SRB the principal group of microorganisms linked to the anaerobic corrosion of metal surfaces due to the generation of sulphides that are extremely corrosive, toxic, and reactive [4,5]. In fact, SRB are responsible for roughly half of all MIC cases, posing both environmental and industrial hazards [6]. Thus, there is a pressing necessity for ultra-sensitive, specific detection methods which would help in corrosion analysis and environmental monitoring.

Traditional microbiological tests for the identification of SRB are often time-consuming and challenging [2]. Considerable attention has been focused on developing rapid and reliable detection methods of SRB in both natural and industrial settings [7]. Different methods are employed to identify SRB in environmental samples, categorized into direct detection and culture methods. Traditional culture techniques are slow due to bacterial growth [8] and often fail to accurately characterize the diversity and abundance of bacteria present in the sample [9,10]. In contrast, direct detection methods employing biomarkers such as adenosine triphosphate (ATP), nucleic acids (RNA and DNA), proteins, and metabolic products offer insights into community structure [11], but these approaches remain under development and have to face different challenges when used in situ [12]. On the other hand, metagenomics, involving large-scale DNA sequencing directly from environmental samples, allows for the genomic investigation of microbial communities without prior cultivation in the laboratory [13]. Polymerase chain reaction (PCR) is an extensively employed approach for SRB detection, offering simplicity and sensitivity [14]. PCR allows highly sensitive quantification by targeting functional markers like the 16S rRNA gene [15]. However, PCR has drawbacks like a long time requirement and a need for sophisticated equipment to provide the results with selectivity and sensitivity [14].

Recognizing these limitations, the development of new biosensors is in progress [16]. Different types of biosensors have been used to detect sulphate-reducing bacteria, including electrochemical [16,17,18] and optical [19,20,21] biosensors. As an alternative to more classical systems, nanotechnology is a valuable avenue for biosensor development [22]. Particularly, nanomaterials have been employed in the design of gated systems for sensing applications [23,24]. In these sensors, a cargo (generally a dye or fluorophore) is enclosed within a porous support and is selectively released in the presence of a specific analyte [25,26,27,28]. Due to the possibility of using different porous supports such as mesoporous silica nanoparticles [29,30,31] and nanoporous anodic alumina [32,33,34], different gating mechanisms [35,36], and an array of indicator molecules [27,28], this approach is highly flexible. For instance, different oligonucleotide-capped mesoporous materials have been successfully designed in our laboratory for miRNA in patient samples [37,38] and *Mycoplasma fermentans* bacteria in cell culture media [39,40], in addition to *Candida albicans* [41], *Staphylococcus aureus* [42], and *Candida auris* [43] in blood sample detection. More recently, detection methods for SARS-CoV-2 [44,45] and human papilloma virus [46] in clinical samples have also been described using mesoporous gated materials.

Here, we describe a new approach for detecting SRB using gated porous supports. In particular, we are focused on the detection of *Desulfovibrio vulgaris* due to its relevance in the field of the metabolism and biochemistry of SRB [47]. Furthermore, it was the first SRB genome sequenced [48], which further emphasizes its relevance. There are several studies that highlight its important role in biocorrosion and use different genes as molecular markers for its identification [7,49,50].

In our work, the biosensor consists of rhodamine B, used as the fluorescent reporter, loaded into the pores of nanoporous anodic alumina (NAA) materials and coated with a complementary oligonucleotide sequence that specifically binds to a *Desulfovibrio* genome sequence. If *Desulfovibrio* DNA is present, it hybridizes with the capping oligonucleotide, unblocking the pores and releasing rhodamine B. This new approach to detect the presence of *Desulfovibrio* has been tested in aqueous solutions and biological media. Implementing this approach for the detection of *Desulfovibrio* and monitoring water supply networks will allow the real-time control of abnormal bacterial growth inside pipes.

## 2. Materials and Methods

### 2.1. Probe Design

A comprehensive literature review was undertaken to identify effective strategies for DNA probe design targeting SRB (sulphate-reducing bacteria), with a particular focus on the genus *Desulfovibrio* within the class Deltaproteobacteria, due to its relevance in our study. A dataset of 57 sequences of the 16S rRNA gene from the *Desulfovibrio* genus was selected from the NCBI (National Center for Biotechnology Information) platform. Common alignment regions were identified within these sequences for probe construction, and a sequence was generated using the BioEdit Sequence Alignment Editor tool. Target regions from 25 to 60 bp were evaluated based on linearity and the absence of stable secondary structures, such as hairpin loops, that could interfere with the hybridization efficiency of the sensing mechanism. Specificity for *Desulfovibrio* was assessed using BLAST alignment [51], while structural stability was analyzed using the UNAFold folding algorithm [52].

Following these analyses, the sequence 5′-GAC AGG TGC ATG GCT GTC GTC AGC TCG TGC CGT G-3′ was selected for its high conservation and structural suitability. To help anchor it to the material surface, a poly-T/poly-G tail (TTTTTTGGGG) was added to this sequence for hybridization with a complementary linker strand **O1** (5′-AAA AAA CCC C-3′) linked to the material (*vide infra*). Thus, the sequence **O1** helps the anchoring of the sequence **O2**, which combines both the tail and the probe region (5′-TTT TTT GGG G GAC AGG TGC ATG GCT GTC GTC AGC TCG TGC CGT G G GGG TTT TTT-3′). The oligonucleotide sequences were obtained from Integrated DNA Technologies (Leuven, Belgium).

### 2.2. Chemical Reagents

The reagents utilized throughout this study include 3-(triethoxysilyl)propyl isocyanate, rhodamine B, tris(hydroxymethyl)aminomethane (TRIS), triethylamine (TEA), anhydrous acetonitrile ≥ 99.8% (CH_3_CN), magnesium chloride (MgCl_2_), and hydrochloric acid (HCl), acquired from Sigma-Aldrich (Madrid, Spain). These materials played essential roles in surface functionalization, fluorescent dye encapsulation, and buffer preparation for hybridization and detection assays.

### 2.3. Microscopy and Spectroscopy

A high resolution field emission scanning electron microscopy (HR-FESEM) analysis was conducted with a ZEISS Gemini SEM 500 microscope (Jena, Germany) operated at a voltage of 5 kV. Elemental composition and surface distribution were analyzed by energy-dispersive x-ray spectroscopy (EDXS) utilizing an X-ray detector integrated into the microscope. Fluorescence spectroscopy evaluation was carried out with a BioTek Synergy H1 microplate reader (Winooski, VT, USA).

### 2.4. Fabrication of Oligonucleotide-Capped Materials

The NAA materials were sourced from InRedox^®^ (Longmont, CO, USA) and used as the solid supports designated **S0** (NAA films of 2 mm in diameter, 5 nm in pore diameter, and 10 μm in alumina thickness). **S0** supports were initially loaded with a rhodamine B solution in CH_3_CN (1.57 mM, 8 mL) and incubated for 24 h to facilitate dye encapsulation. The cargo-loading step was followed by the chemical functionalization of the surface via a reaction with 3-(triethoxysilyl)propyl isocyanate (1 mL, 4 mmol), under continuous stirring for 5.5 h to obtain functionalized supports labelled **S1**.

To proceed with probe attachment, **S1** supports were maintained at room temperature during incubation for 3 h in a solution containing rhodamine B in CH_3_CN (1.57 mM, 2.1 mL), **O1** (30 μL, 100 μM), and TEA (12 μL) to obtain intermediate product **S2**. Finally, hybridization with oligonucleotide **O2** (10 μL, 100 μM) was carried out in a hybridization buffer (100 μL, 20 mM Tris-HCl, 37.5 mM MgCl_2_, pH 7.5) at room temperature under stirring for 2 h, leading to the final capped sensor, referred to as **S3**. To remove unbound sequences, the **S3** materials were washed repeatedly with TRIS buffer and subsequently air-dried at 4 °C overnight.

### 2.5. Cargo Quantification

To evaluate dye release capacity, two separate **S3** supports were individually immersed in 1 mL of TRIS buffer. One support was subjected to agitation at 90 °C for 1 h to induce complete pore opening and facilitate the highest dye release, while the other support continued at 37 °C under identical stirring conditions as a control. The concentration of released rhodamine B was determined through fluorescence spectroscopy at 575 nm (λ_exc_ = 555 nm), using a pre-established calibration curve. The experiment for cargo quantification was repeated three times to guarantee reproducibility.

### 2.6. Detection Procedure

To assess detection performance, two groups of **S3** supports were incubated in 900 µL of TRIS buffer. One group received 100 μL of the complementary **O2c** strand (1 μM, 13.8 ng·μL^−1^), while the other was supplemented with an equivalent volume of TRIS buffer as a control. Fluorescence measurements at 575 nm (λ_exc_ = 555 nm) were taken every 15 min over a 1 h period at 37 °C to monitor dye release kinetics.

In a parallel experiment, the same procedure was repeated using genomic DNA (1.8 ng·μL^−1^) extracted from *Desulfovibrio vulgaris* (DSM 644, DSMZ, The Leibniz Institute DSMZ—German Collection of Microorganisms and Cell Cultures GmbH, Braunschweig, Germany), replacing the synthetic oligonucleotide **O2c**.

The next step was testing the system’s capacity to detect bacterial DNA from cell culture samples. Thus, a diluted culture of *Desulfovibrio* with a known concentration of 10^3^ cells·μL^−1^ was prepared in TRIS buffer. As before, two sets of **S3** materials were prepared for evaluation, and both were immersed in 900 μL of TRIS buffer. One was treated with 100 μL of the diluted bacterial sample, and 100 µL of TRIS buffer was added to the other. After 1 h of incubation at 37 °C, fluorescence emission from delivered rhodamine B was measured (λ_exc_ = 555 nm) to determine target-induced pore opening.

### 2.7. Sensitivity Analysis

The detection sensitivity of the system was evaluated by analyzing the reaction of individual **S3** materials to serial dilutions of the *Desulfovibrio* culture, with concentrations ranging from 0.01 to 10^8^ cells·mL^−1^. Each concentration was tested using a separate support, and 100 µL of bacterial suspension was introduced to 900 µL of TRIS buffer to reach a final volume of 1 mL. The mixtures were incubated at 37 °C for 1 h under gentle agitation. Fluorescence emission at 575 nm (λ_exc_ = 555 nm) was measured to quantify the rhodamine B released in response to each concentration.

### 2.8. Selectivity Analysis

To assess the selectivity of the sensor, the same protocol described previously was employed in the presence of different non-target bacterial species, including *Escherichia coli* (10^3^ cells·mL^−1^), *Staphylococcus aureus* (10^3^ cells·mL^−1^), *Staphylococcus epidermidis* (10^3^ cells·mL^−1^), and *Lactobacillus rhamnosus* (10^3^ cells·mL^−1^). For each species, 100 µL of bacterial suspension was added to 900 µL of TRIS buffer containing the **S3** material. In addition, two groups were included: a positive control group with *Desulfovibrio* (10^3^ cells·mL^−1^) and a negative control group containing only TRIS buffer. After 1 h of incubation at 37 °C, rhodamine B fluorescence was evaluated at 575 nm (λ_exc_ = 555 nm).

### 2.9. Testing System’s Performance in Water Samples

To evaluate the performance of the detection platform under realistic conditions, an assay was conducted using water samples collected from the metropolitan water network of Valencia. The performance of the **S3** supports was evaluated in TRIS buffer solutions containing varying proportions of tap water. For each condition, two sets of **S3** solids were submerged in 900 µL of the corresponding buffer/water mixture. One was treated with 100 μL of the complementary **O2c** strand (13.796 ng·μL^−1^), while the other received 100 µL of TRIS buffer only. After 1 h of stirring at 37 °C, the released rhodamine B was measured by fluorescence at 575 nm (λ_exc_ = 555 nm).

The same procedure was carried out again with water-diluted bacterial cultures at concentrations ranging from 10 to 10^4^ cells·mL^−1^ to verify system functionality in complex aqueous environments.

## 3. Results and Discussion

### 3.1. Fabrication and Characterization of Sensors

**Figure 1:**
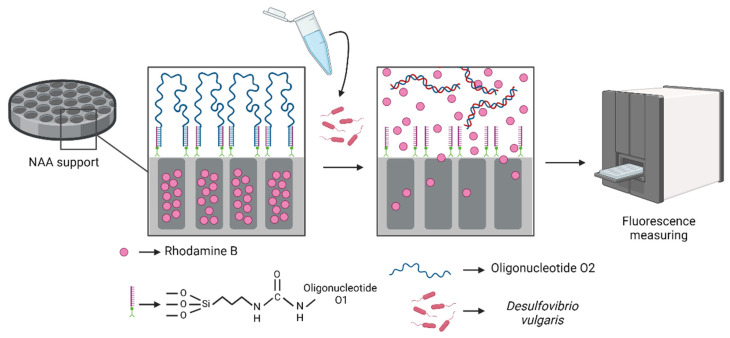
Scheme of the controlled release system. The sensor **S3**, loaded with rhodamine B and capped with the **O2** sequence, delivers the encapsulated rhodamine B dye in response to the target DNA from *D. vulgaris*.

The production of the sensing platform involved a multi-step procedure. Initially, nanoporous anodic alumina materials (**S0**) were filled with rhodamine B through passive diffusion using a concentrated solution of the dye in acetonitrile. Subsequently, the surface underwent chemical modification with 3-(triethoxysilyl)propyl isocyanate, turning into an intermediate **S1** solid. In the next step, an amine-functionalized oligonucleotide (**O1**) was covalently attached via urea bond formation, obtaining **S2**. Final pore capping was achieved by hybridization between linker **O1** and sequence **O2**, resulting in the final sensor **S3**, where the pores were effectively sealed. Upon exposure to *Desulfovibrio* DNA, specific hybridization with the capping strand triggered pore opening and rhodamine B release to the medium (Figure 1).

**Figure 2:**
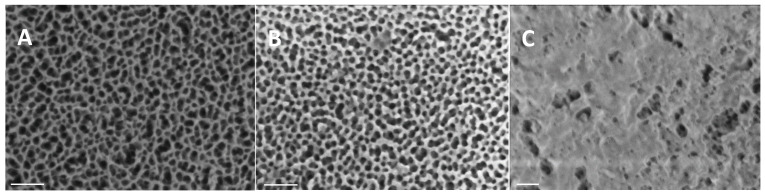
HR-FESEM images of NAA supports during the fabrication process (scale bars: 100 nm). (**A**) NAA plate without modification (**S0**). (**B**) Loaded and functionalized NAA plate with rhodamine B and isocyanate (**S1**). (**C**) Loaded, functionalized, and capped NAA plate with **O2** (**S3**).

The characterization of each stage (**S0**–**S3**) was conducted by HR-FESEM and EDXS analysis to verify that the different steps were successfully modified. Solids **S0**, purchased from commercial sources, consisted of anodic aluminum oxide films atop a 0.1 mm thick aluminum base with a density of 9·10^11^ pores∙cm^−2^ (Figure 2). According to the supplier specifications, the pore morphology was conical, with diameters ranging from 20 to 30 nm at the opening and narrowing to 5 nm at the base. This structure achieved a higher level of uniformity along a 10 µm length at a depth of 15 nm. No significant morphological differences were observed between solids **S0**, **S1**, and **S2**, indicating that surface functionalization did not alter the structure’s integrity. However, **S3** displayed a distinct surface coating, attributed to the immobilized **O2** strands, which confirmed successful pore closure.

**Figure 3:**
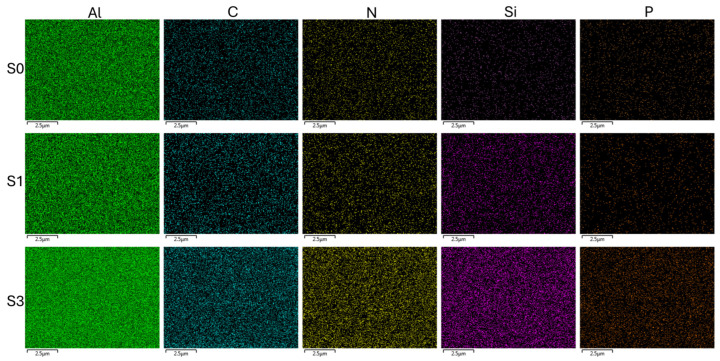
Elemental mapping images of the support surfaces for samples **S0**, **S1**, and **S3**, obtained by energy-dispersive X-ray spectroscopy (EDXS). The spatial distribution of key elements (Al, C, N, Si, and P) is shown to illustrate the progressive surface modification throughout the functionalization process.

The elemental atomic analysis of the supports’ surfaces was determined employing energy-dispersive X-ray spectroscopy (EDXS) to complement HR-FESEM imaging and confirm changes in the surface composition throughout the sensor fabrication process. As shown in Table 1, a progressive increase in C and Si atomic content from supports **S0** to **S1** was detected, confirming the successful incorporation of rhodamine B and functionalization with the alkoxysilane groups, respectively. Moreover, phosphorous was identified in solids **S3**, resulting from the phosphate backbone of the capping oligonucleotide **O2**. In addition, representative elemental mapping images were obtained to further support these findings. As shown in Figure 3, the spatial distribution of key elements across the material surface can be observed. The phosphorus signal, which is lightly present in samples **S0** and **S1**, becomes clearly detectable only in **S3**, confirming the successful immobilization of the oligonucleotide. This observation is consistent with the elemental analysis data presented in Table 1, as well as with the HR-FESEM images, which show a surface coating indicative of pore closure. Together, these results support the effective functionalization and chemical modification achieved in the final stage of material preparation.

Finally, quantification experiments revealed that the maximum concentration of rhodamine B contained in solids **S3** was 26.6 ng per g of material.

### 3.2. Release Assays

After the fabrication of the supports, release assays in the presence of the DNA sequence of *Desulfovibrio* **O2c** complementary to the capping oligonucleotide were carried out. This study allowed for the evaluation of the specific release of the cargo due to the recognition between the complementary **O2c** and the capping oligonucleotide. For this assay, two different sets of gated supports were employed and submerged in TRIS buffer. In total, 100 μL of the complementary *Desulfovibrio* sequence **O2c** (1 μM) was added only to one of the solids, whereas 100 μL of TRIS buffer was added to the other one. The release of dye into the aqueous solution was observed by taking periodic aliquots every 15 min for 1 h at 37 °C, and the fluorescence was measured at 575 nm (λ_exc_ = 555 nm). The obtained results indicate that the **S3** control supports incubated in TRIS buffer showed a negligible rhodamine B release, even at longer times, supporting the capping efficiency of oligonucleotide probe **O2** that blocked the pores and avoided rhodamine B release. However, high fluorescence enhancement was observed from **S3** when exposed to the complementary oligonucleotide that unblocked the pores, leading to dye delivery.

**Figure 4:**
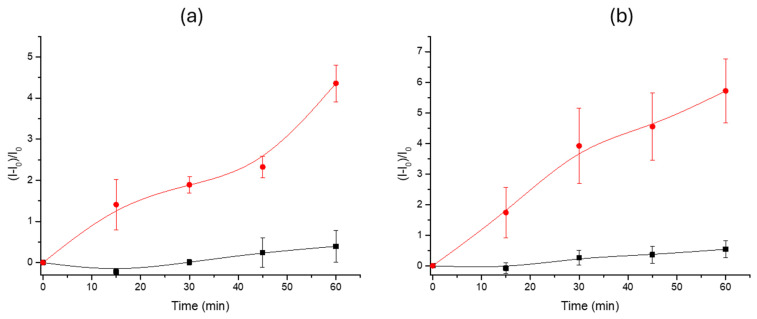
Fluorescence emission kinetics of released RhB from supports **S3** in control media TRIS solution with *Desulfovibrio vulgaris* (**a**) genomic DNA and (**b**) cell culture sample. The red line shows the release profile in the presence of *D. Vulgaris* gDNA or cells, and the black line represents the release in the absence of DNA or the bacteria. Data are expressed as mean values and standard deviations derived from measurements for 3 distinct supports.

Once the functionality of the sensor platform with the specific *Desulfovibrio* DNA sequence was evaluated, the system behaviour was tested using the whole extracted genomic DNA. Like in the previous experiment, two independent sets of **S3** solids were immersed in TRIS solution. In total, 100 μL of the gDNA of *D. vulgaris* (1.8 ng·μL^−1^) was added to one support, and 100 μL of TRIS buffer was added to the other one, which acted as a control assay. Supports were incubated in their respective solution for 1 h at 37 °C, and periodic aliquots of the supernatant were taken every 15 min to measure fluorescence at 575 nm (λ_exc_ = 555 nm). Results are summarized in Figure 4a. A higher rhodamine B release was observed from the supports that were in the presence of the bacterial gDNA compared to the delivery from the control supports in the presence of TRIS buffer solution. This procedure confirmed the system’s capacity to identify and react to the presence of *D. vulgaris* DNA, thereby verifying its effectiveness.

The subsequent step in the evaluation of the system involved an assessment of the capability to detect the bacteria’s DNA directly from a bacterial culture sample. To accomplish this test, a sample of *D. vulgaris* culture resuspended in TRIS solution with a known concentration of 10^3^ cells·mL^−1^ was used. As in previous experiments, two sets of S3 supports were needed. Both sets were submerged in 900 µL of TRIS medium, and 100 µL of the diluted bacterial culture was added to one of the sets, while 100 µL of TRIS medium was added to the other set of supports. Following a 1 h incubation at 37 °C, the amount of free rhodamine B was determined by measuring fluorescence emission at 575 nm (λ_exc_ = 555 nm). The results obtained are shown in Figure 4b. A higher fluorescence emission was detected from the supports that were in medium with bacterial culture. These results point out that sample pretreatment is not necessary for the detection of the bacteria’s DNA. The effect of TRIS buffer to enhance cell membrane permeability has been described in several works [53,54,55,56] and could explain the direct detection of the bacteria’s DNA without the previous heating or lysis treatment of the samples.

### 3.3. Sensitivity Assay

**Figure 5:**
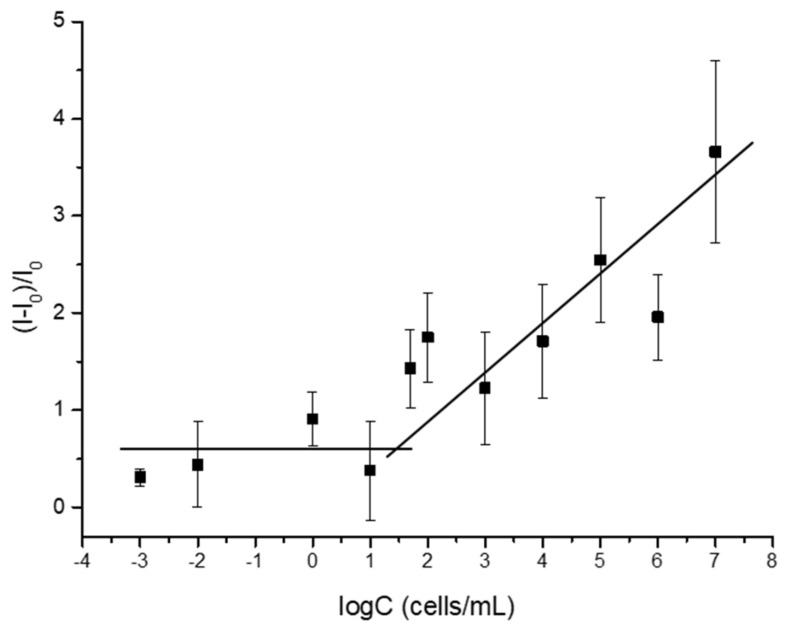
Rhodamine B release in response to increasing concentrations of *D. vulgaris* cell culture.

The system’s sensitivity was assessed by studying the materials’ behaviour to a range of concentrations of *D. vulgaris* culture. Thus, eleven separated supports were immersed in TRIS buffer with different bacterial concentrations spanning from 0.001 to 10^7^ cells·mL^−1^. After a 1 h incubation period at 37 °C, the fluorescence of the released rhodamine B from the pores was measured at 575 nm (λ_exc_ = 555 nm). The results shown in Figure 5 reveal that fluorescence emission exhibited a direct linear correlation with the concentrations (C) of *D. vulgaris* cells in the 10–10^7^ cell mL^−1^ range. Thanks to the cargo quantification assay, we can calculate the percentage of rhodamine B released with the bacterial samples. We use the fluorescence signal obtained under forced pore-opening conditions at 90 °C as the reference for the 100% cargo release capacity of the sensor. Based on this, we determined that 42.6% of the total loaded dye was released after a 1 h incubation with *D. vulgaris* cells at the highest concentration used (10^7^ cells·mL^−1^), while only 3.17% of the total amount of entrapped rhodamine B was released at lower concentrations of cells (50 cells·mL^−1^). Moreover, the limit of detection (LOD) of the system was established by locating the intersection point of the horizontal line and the positive slope line. Following this approach, the LOD was calculated to be 30 cells·mL^−1^. This LOD is similar to other values previously reported for SRB detection using different sensors, such as the plastic optical fibre immunosensor [19] and a CdS nanoparticle-based fluorogenic sensor [20]. This result shows high sensitivity of the oligonucleotide-gated nanoporous alumina-based sensors **S3** for the detection of *D. vulgaris*.

Furthermore, it is interesting to note that the sensing system shows a remarkable amplification, as it releases a substantially greater amount of entrapped rhodamine B molecules in comparison to the quantity of *D. vulgaris* cells needed to produce this release. The number of dye molecules released compared to the number of cells at concentrations near the LOD (50 cells·mL^−1^) was calculated to be around 5·10^10^ RhB molecules released per cell, showing a great signal amplification that is similar to previously obtained data in other systems based on the same technology [42].

### 3.4. Selectivity Analysis

**Figure 6:**
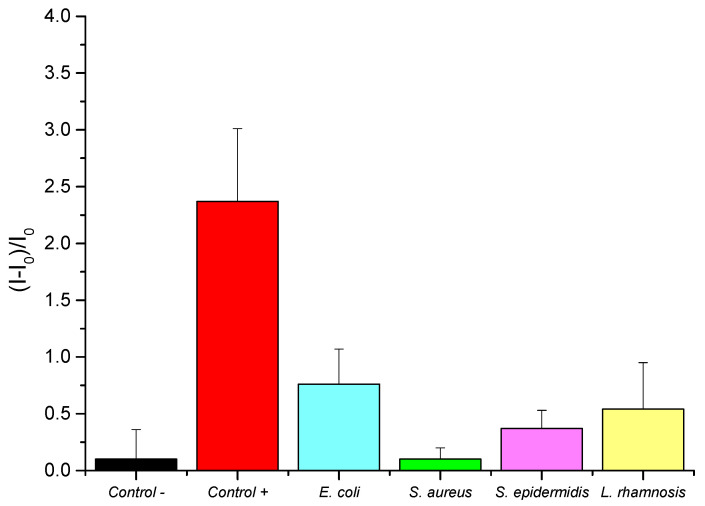
Interference assay: dye release in the presence of different bacterial species, including *E. coli*, *S. aureus*, *S. epidermidis*, *L. rhamnosus*, and *D. vulgaris* cell cultures.

The selectivity of the biosensor was studied by evaluating the behaviour of S3 to different bacterial species, including *E. coli*, *S. aureus*, *S. epidermidis*, and *L. rhamnosus*. *E. coli* is the main interfering pathogen in *Desulfovibrio* detection because both are Gram-negative bacteria and could be found in the same samples. Selectivity studies involved testing independent S3 supports with each bacterial culture sample, each set diluted with TRIS buffer to the same final concentration (10^3^ cells·mL^−1^). For each assay, 100 μL of the corresponding culture sample were introduced into 900 μL of hybridization buffer, where S3 solids were previously immersed. In addition, two other sets of supports were included in this study: a positive control group with a *D. vulgaris* culture sample and a negative control group with TRIS buffer. Then, supports were stirred at 37 °C for 1 h, and the release of rhodamine B was quantified by measuring the fluorescence emission at 575 nm (λ_exc_ = 555 nm). Results are summarized in Figure 6. The released rhodamine B from sensors in contact with other bacterial species was similar to the delivered dye from control sensors. Moreover, the highest fluorescence signal was obtained from S3 biosensors in the presence of the *D. vulgaris* cells, supporting the selectivity of the oligonucleotide-gated nanoporous sensor for the detection of the *D. vulgaris* target cell DNA.

The residual fluorescence from *E. coli* and *L. rhamnosus* may result from partial sequence similarity in the non-target genome or minor non-specific hybridization events, though these are not sufficient to generate a false-positive signal. Despite these background signals, the fluorescence emission from *D. vulgaris* remains at least three times higher than any of the fluorescence produced from non-target bacteria, demonstrating effective detection.

### 3.5. Performance Analysis in Inoculated Water Samples

After the characterization of the materials in control media (TRIS buffer), the system’s robustness for the detection of *Desulfovibrio* in real media was conducted using water samples obtained from the Valencia water network. First, the behaviour of the supports was evaluated by employing a range of percentages of TRIS buffer and water sample in the final mixtures. For each condition, two independent sets of **S3** solids were submerged in 900 µL of TRIS/water mixture, and to one of them, 100 µL of the **O2c** sequence was added, and TRIS solution was added to the other one. The solution was agitated for 1 h at 37 °C. Subsequently, the released rhodamine B was quantified through fluorescence measurement at 575 nm (λ_exc_ = 555 nm). The best result was accomplished using a mixture with 25% water. A greater addition of water to the media affected the stability and the performance of the system.

**Figure 7:**
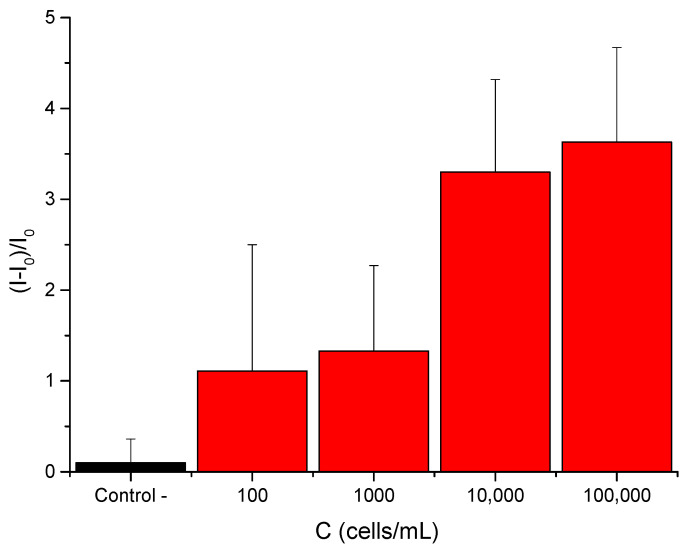
Fluorescence emission from support **S3** in TRIS/water samples containing *D. vulgaris* cells. The red bars show the release in the presence of *D. vulgaris* cells from bacterial cultures at different concentrations, and the black bar represents the release in the absence of the bacterial cells.

In a further step, the biosensor’s response using different concentrations of *Desulfovibrio* bacterial culture in the optimal TRIS/water mixture using a 25% water sample was evaluated. Five sets of **S3** solids were immersed in 900 µL of the TRIS/water mixture. In total, 100 µL of TRIS buffer was added to the control support, whereas 100 µL of bacterial culture was added to the other supports in the concentration (C) range of 10^2^–10^5^ cells·mL^−1^. After 1 h, the quantity of released rhodamine B was measured through the fluorescence emission at 575 nm (λ_exc_ = 555 nm). Results can be observed in Figure 7. The supports incubated with cell samples (red bars) exhibited enhanced fluorescence emission intensities in comparison to the control sample (black bar). These preliminary results prove the sensor’s capacity to detect *D. vulgaris* cell DNA in mixtures of water samples and TRIS buffer with high sensitivity and selectivity, supporting its potential use for the in situ control of MIC in water supply networks.

## 4. Conclusions

An effective sulphate-reducing bacteria detection platform integrating nanostructured hybrid organic–inorganic materials with the technology of molecular gates has been successfully developed. This system relies on nanoporous anodic alumina films with a fluorescent dye entrapped and gated with a specific oligonucleotide probe, creating a platform for the detection of *Desulfovibrio* DNA. In the system, the detachment of the oligonucleotide gate occurs when the complementary sequence of *Desulfovibrio* DNA is present. This event induces the release of rhodamine B from the porous structure, which was detected by a fluorescence read-out. To ensure the robustness and reliability of the detection system, the surface morphology of the sensors was meticulously characterized using high-resolution field emission scanning electron microscopy. Additionally, the surface atomic composition was calculated by energy-dispersive X-ray spectroscopy, providing a comprehensive understanding of the sensor’s structural integrity and chemical composition. The biosensor’s capacity to detect the genomic DNA of *D. vulgaris* and the DNA of bacterial cells in culture samples was demonstrated. Sensitivity was proven at different concentrations of the cell culture, achieving a limit of detection of 30 cells ml^−1^, and selectivity was demonstrated in the presence of different bacterial samples *of Escherichia coli*, *Staphylococcus aureus*, *Staphylococcus epidermidis*, and *Lactobacillus rhamnosus*. The sensor’s performance for the detection of *D. vulgaris* cell DNA was further validated in real media, using water samples from the Valencia water network. The best result to detect *Desulfovibrio*’s DNA in water samples was obtained using a mixture of 75% TRIS buffer and 25% water sample. This successful validation underscores the system’s potential use for the in situ control of microbiologically influenced corrosion in water supply networks. This technology represents a significant advance in the field of sulphate-reducing bacteria detection, offering a versatile and reliable solution for directly monitoring this bacterial group in water networks without any sample pretreatment. The integration of nanostructured materials and molecular detection techniques positions this system as a valuable tool for ensuring the safety and integrity of water supplies, particularly in regions susceptible to MIC.

## Figures and Tables

**Table 1:** Elemental atomic composition of S0, S1, and S3.

	C/Al	N/Al	Si/Al	P/Al
S0	0.14 ± 0.01	-	-	-
S1	0.48 ± 0.10	0.07 ± 0.02	0.10 ± 0.04	-
S3	0.35 ± 0.01	0.05 ± 0.01	0.04 ± 0.02	0.04 ± 0.01

## Data Availability

The original contributions presented in this study are included in the article. Further inquiries can be directed to the corresponding author.

## Abbreviations

The following abbreviations are used in this manuscript:

MIC	Microbiologically influenced corrosion
SRB	Sulphate-reducing bacteria
ATP	Adenosine triphosphate
RNA	Ribonucleic acid
DNA	Deoxyribonucleic acid
PCR	Polymerase chain reaction
NAA	Nanoporous anodic alumina
